# Diagnostic dilemma in a patient with upper arm mass with history of breast cancer and melanoma

**DOI:** 10.1259/bjrcr.20210218

**Published:** 2022-11-01

**Authors:** Hanna Wyciszczok, Nuala Ann Healy, Helen Taylor

**Affiliations:** 1 Cambridge University Hospitals NHS Foundation Trust, Cambridge, UK; 2 Newnham College University of Cambridge, Cambridge, UK

## Abstract

We present a case of upper limb muscle metastasis in a female patient with a history of malignant melanoma. Although melanoma is the fifth most common cancer in the UK, muscle metastases are extremely rare, with only a few cases reported in the literature. We discuss the challenge of diagnosing muscle metastasis on radiological imaging and in particular of distinguishing metastatic lesions to muscle from sarcoma. We also review the imaging findings of other published cases in a literature review. We conclude that although certain characteristic features of melanoma metastases can be identified on imaging (*e.g.,* hyperintensity on *T*_1_-weighted MRI), the radiological appearances are highly variable and histopathological examination is necessary to confirm the diagnosis.

## Case history

### Clinical history

An 83-year-old female was referred by her General Practitioner to our symptomatic Breast Clinic with a right upper arm mass. She was noted to have significant past medical history of right breast cancer and melanoma. Her breast cancer was diagnosed and treated with right mastectomy in 2013 and she continues to take tamoxifen as adjuvant treatment. She was first diagnosed with melanoma in 2015, when she presented with an enlarging and non-healing skin lesion on her right forearm. She was referred to dermatology for urgent excision. Histology demonstrated the lesion to be a superficial spreading malignant melanoma 4.4 mm Breslow thickness with ulceration. She was further treated with wide local excision of the scar on the right forearm. Staging CT of head, chest, abdomen and pelvis showed no evidence of metastatic disease at that time. The option of sentinel node biopsy was offered and discussed; however the patient declined to proceed with this procedure.

In 2016, she presented with a right axillary mass which was biopsied and confirmed to be metastatic melanoma. Right axillary lymph node dissection was performed and histopathology confirmed that three out of twelve nodes contained melanoma; however there was no extracapsular extension. The melanoma Multidisciplinary Team (MDT) recommended adjuvant radiotherapy, however after discussion of risks and benefits patient opted not to have this treatment.

**Figure 1.**
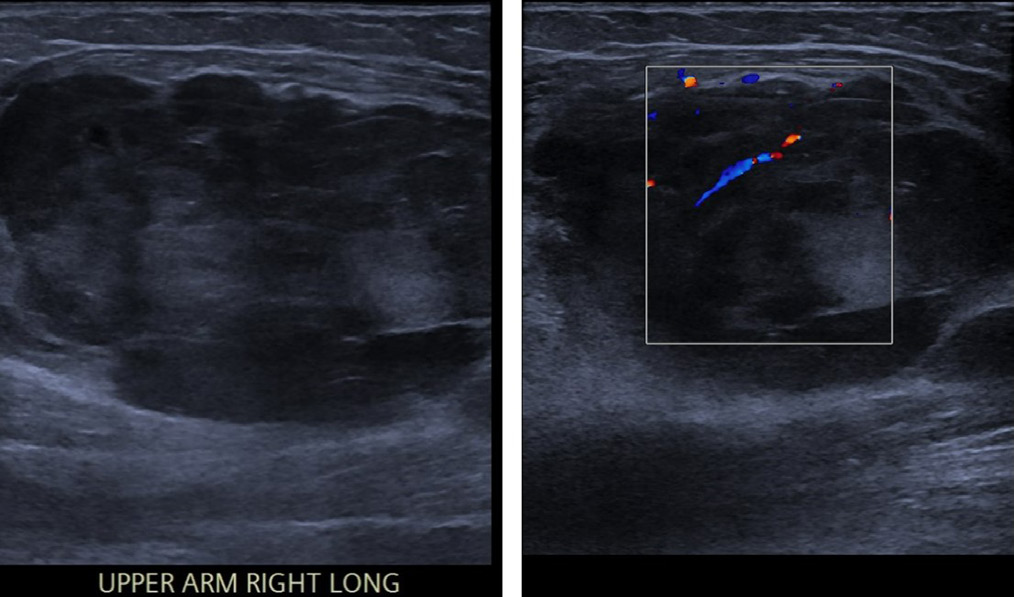
US right axilla and upper arm revealed a 42 × 37 × 70 mm soft tissue intramuscular mass in the right biceps. This was relatively encapsulated and demonstrated marked mixed internal echogenicity and internal vascularity.

For the current presentation, the patient described a 2–3 month history of right upper arm swelling, associated with right arm and shoulder pain. She also reported three stone weight loss (approx. 19 kg) over the preceding 12 months. Clinical examination revealed a palpable large right upper arm mass related to the biceps muscle. Mammogram of the left breast was normal. Ultrasound of the right axilla and right upper arm did not demonstrate any abnormal axillary lymph nodes but did reveal an intramuscular soft tissue mass involving the right biceps muscle (Figure 1).

### Differential diagnosis

This case posed a diagnostic challenge given the patient’s previous medical history of two malignancies (previous right forearm melanoma and right sided breast cancer). The differential diagnosis of the right arm mass included both primary soft tissue lesion (*i.e.,* sarcoma) and metastatic disease. Due to the potential diagnosis of sarcoma and risk of seeding, the mass was not biopsied at initial presentation and the patient was referred for further imaging including MRI and staging CT thorax/abdomen/pelvis.

### Imaging findings

**Figure 2.**
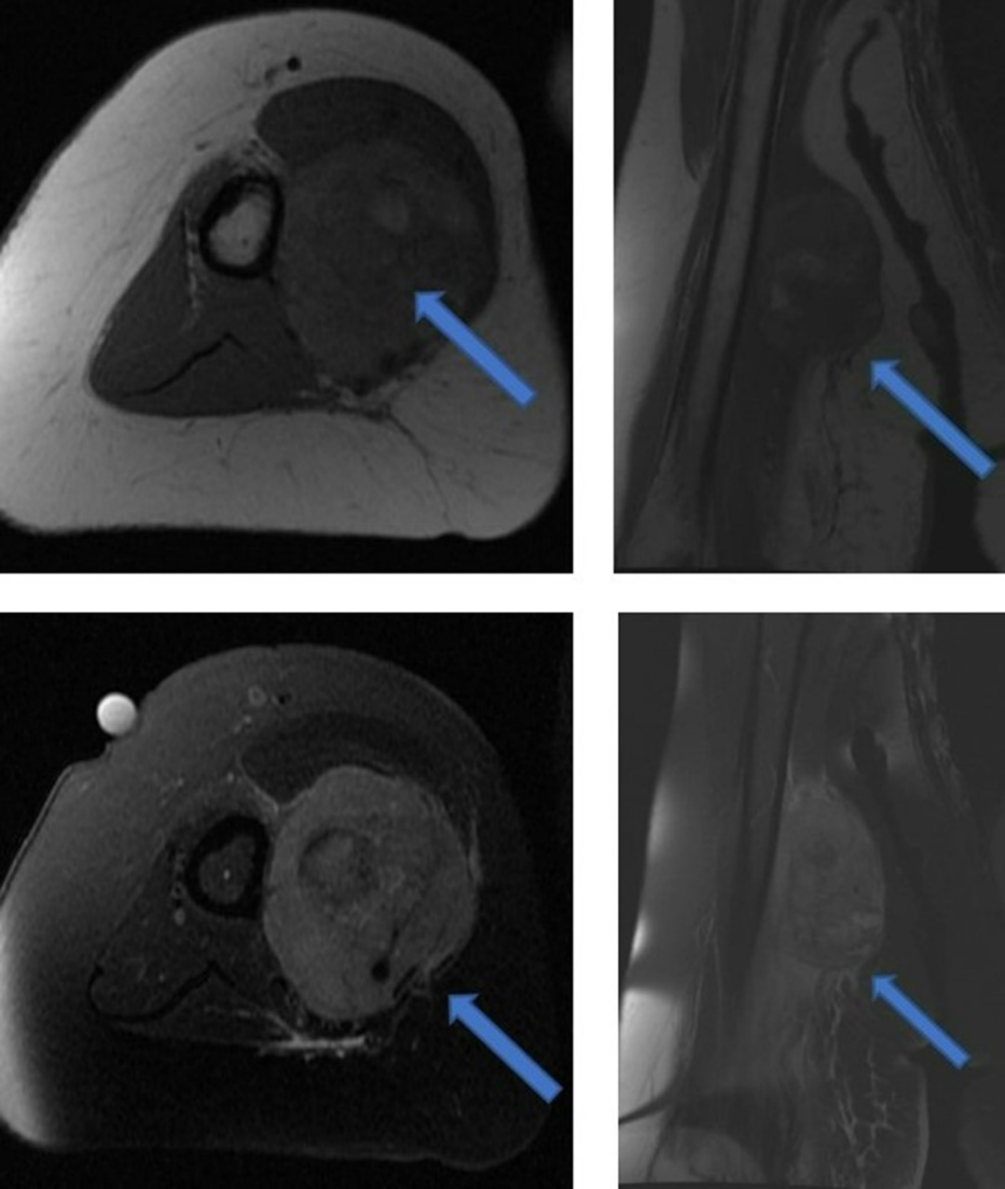
MRI of right upper arm lesion (arrows). Figure 2a and b are *T_1_*-weighted axial and coronal sequences demonstrating high signal centrally suggestive of intralesional haemorrhage. Figure 2c and d are *T_2_* fat-saturated sequences illustrating perilesional oedema with no periosteal reaction or underlying bone lesion.

MRI showed a deep soft tissue lesion involving the anterior compartment of the right arm measuring 50 × 45 × 65 mm at the level of the mid humerus (Figure 2). The lesion was mildly hyperintense to the adjacent skeletal muscle on both *T_1_*- and *T*_2_-weighted sequences and was found to contain central *T_1_* high signal areas suggestive of intralesional haemorrhage. There was extensive peri-lesional muscular oedema, but no periosteal reaction, cortical breach, or marrow replacement of the underlying right humerus. The findings were in keeping with an aggressive soft tissue lesion of the right upper arm, concerning for malignancy.

**Figure 3.**
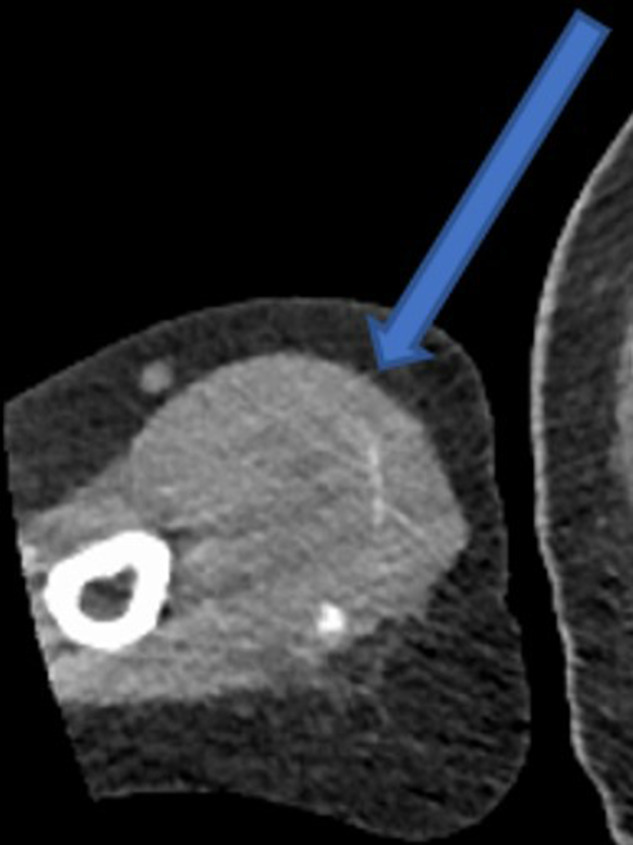
Axial CT image showing muscle lesion in the right arm (arrow).

A subsequent staging CT was performed and showed no evidence of further metastatic lesions; the right arm mass was visualised on axial imaging (Figure 3).

The patient was discussed at the sarcoma MDT, who referred her for ultrasound-guided biopsy. Histopathology of the lesion revealed a diagnosis of metastatic melanoma. Following this, the case was discussed at the melanoma MDT who recommended surgical management and adjuvant treatment with immunotherapy or radiotherapy.

## Discussion

Melanoma is the fifth most common cancer in the UK, affecting around 16,200 people each year, with increasing prevalence over the last few decades.^1^ However, it is very uncommon to see skeletal muscle metastasis in melanoma, especially to the upper limb, with only a few cases reported in the literature. Melanomas most frequently metastasise to skin, subcutaneous tissues, and distant lymph nodes, after which the next most frequent sites are the brain, liver, and bone.^2^ In fact, muscle metastases from any solid tumour are rare, and most frequently affect the thigh muscles, the extraocular musculature, and the gluteal and paravertebral muscles.^3^

The underlying mechanism of why muscle metastases are so rare is still unclear. Studies suggest that incidence of skeletal muscle metastases from various malignancies ranges from 0.8 to 16%, however those figures are not specific to melanoma primary.^4^ Factors such as highly variable blood flow or high concentration of lactic acid have been suggested to limit growth of metastases in skeletal muscle.^5,6^

Furthermore, autopsy studies^4^ suggest that skeletal muscle metastases occur late in the malignant disease, *i.e*. when multiple other metastases are also present. This might contribute to their reduced detection rate (*e.g.,* in patients under palliative care who do not want to undergo further imaging). Furthermore, a proportion of patients with malignant disease may not survive long enough for these late muscle metastases to develop.

In addition to being rare, muscle metastases can present with a broad spectrum of radiological features, generating additional difficulty in making the diagnosis.

### Appearance of melanoma metastases on MRI

Although definitive diagnosis of metastatic melanoma requires biopsy and histopathological examination,^7^ literature suggests that certain characteristic features of melanoma metastasis can be observed on imaging, particularly on MRI. However, the evidence is sparse given how uncommon these metastases are, with only a few case reports which we review below.

The evidence suggests that melanoma and melanoma metastases commonly appear hyperintense on *T*_1_-weighted MRI.^8^ Two mechanisms underlying this radiological appearance have been proposed: 1) paramagnetism of stable free radicals in melanin and 2) methemoglobin in nonacute haemorrhage of the tumour.^9^

Several case reports report this finding. Yoshioka et al^10^ reported a case of intramuscular metastasis in the lower limb from malignant melanoma, where the lesion appeared as intermediate to high signal intensity on *T*_1_-weighted images and mixed signal intensity on *T*_2_-weighted imaging, containing both high and low signal. Another case report of melanoma metastasising to the lower limb musculature was described by Viswanathan et al,^5^ where again MRI showed a necrotic lesion appearing as heterogenous *T_1_*-weighted imaging with enhancement on post contrast sequences, and hyperintense on *T_2_*-weighted imaging. Dalle Carbonare et al^11^ described a patient with melanoma of unknown primary, whose presenting complaint was a temporalis muscle lesion, which appeared as a lobulated high signal mass on *T_1_*-weighted imaging with no evidence of intralesional fat, with diffusely low signal on *T_2_*-weighted imaging. A further example a *T_1_*-hyperintense lesion was described by Pirlamarla et al^12^ who reported a case of an elderly female with recurrent vulvar melanoma with extraocular muscle metastasis, appearing as a heterogenous hyperintensity on *T*_1_-weighted images of orbital MRI, with high signal on *T_2_*-weighted sequences.

However, the *T_1_*-hyperintense appearance of melanoma metastases is not universal and several other case reports suggest wide variability in radiological findings.^13^ Kuo-Feng Hsu et al^14^ reported a case of scalp melanoma re-presenting with metastasis to rectus abdominis muscle after a nearly 5-year disease-free period – a timeframe similar to the patient in this report. However, the lesion described by Kuo-Feng Hsu et al was hypointense on *T*_1_-weighted image and hyperintense on *T*_2_-weighted image, showed contrast enhancement, and demonstrated restricted diffusion. The patient was treated with surgery but passed away six months later due to development of multiple metastases.

Melanoma metastases can also demonstrate a heterogenous appearance, for example Shih et al^15^ reported a case of ocular muscle metastasis from melanoma primary, which on MRI appeared as a multicystic mass with layering fluid-fluid levels.

The examples presented above illustrate the variability in imaging appearances of melanoma muscle metastases on MR imaging, thus contributing to significant diagnostic difficulty.

### Appearance of melanoma metastases on CT

The evidence seems to be even more sparse for CT appearances compared to MRI, with only a few studies found in the literature.

Two retrospective cohort studies suggest that muscle metastases from any primary most commonly appear as rim-enhancing intramuscular lesions with central hypoattenuation.^16,17^ However, none of the primary malignancies included in those studies were melanomas.

Only a few of the case reports reporting metastatic melanoma to muscle included CT findings. Weiss et al^18^ reported a case of amelanocytic melanoma spreading to extraocular muscles, where CT showed spindle-like swelling of several muscles in the orbit. In the case described by Viswanathan et al,^5^ CT showed a mass in the quadriceps muscle with an enhancing rim and low attenuation centre. In the case reported by Dalle Carbonare et al,^11^ CT showed a well-defined subcutaneous enhancing soft tissue mass in the right temporal fossa.

### Differentiating muscle metastasis and sarcoma

The variability in radiological appearances of muscle metastases contributes to the difficulty in distinguishing a metastasis from primary soft tissue tumour (*e.g.,* sarcoma). One retrospective study found that there were no radiographic characteristics in any imaging modality that allowed differentiation between metastases to muscle and a primary soft tissue sarcoma.^19^ This diagnostic challenge clearly impacts patient care, as the biopsy approach would be different in case of sarcoma compared to muscle metastasis – the current UK guidelines^20^ recommend that a mass suspicious for a soft tissue sarcoma should be investigated with a core needle biopsy (as opposed to fine needle aspiration), planned in a way that the biopsy tract can be safely excised at the time of definitive surgery to reduce the risk of seeding.

The challenge of diagnosing muscle metastasis is further complicated by the fact that well-differentiated liposarcomas can appear hyperintense on *T_1_*, *i.e*. similar to melanoma metastases. However, due to their fat content, the hyperintense area of a liposarcoma would become suppressed on fat-suppressed *T*_1_-weighted images.^8^ Additional difficulty in reaching the diagnosis arises from the fact that melanoma might undergo de-differentiation with loss of melanocytic markers, potentially leading to altered appearances on radiological imaging.^21^ Furthermore, malignancies can co-exist and history of malignant melanoma does not preclude a co-existing sarcoma.^22^

### The role of PET/CT in metastatic melanoma

Increased FDG-18 uptake in skeletal muscle metastasis imaged with FDG PET/CT has been reported by Gómez-León et al,^4^ although this was observed in only one of the analysed cases. The authors also describe increasing use of FDG PET/CT as the modality of choice in initial staging and re-evaluation of metastatic melanoma. We note this modality could potentially have been considered in the patient described in this report to aid diagnosis of any other metastases or lymph nodes, including potential alternative biopsy sites. Indeed, a meta-analysis of literature^23^ reported that a PET/CT was found to be superior to CT, PET and US for the detection of distant metastases in both the staging and surveillance of melanoma patients. Furthermore, staging in patients with disseminated melanoma to assess extent of disease prior to treatment is one of the evidence-based indications for performing PET/CT according to the Royal College of Radiologists guidance.^24^

However, PET/CT is not recommended by the current NICE guidelines as the modality of choice for investigating suspected metastatic melanoma.^25^ Although these guidelines report a sensitivity and specificity of PET/CT for the identification of any metastases in melanoma of 90.6% and 77.2%, this was based on only one study and this modality was not included in the recommendations. Instead, the guidelines recommend CT staging to people with stage III or suspected stage IV melanoma.

## Clinical outcomes

As mentioned above, skeletal muscle metastases usually occur late in the malignant disease, and often the presence of such lesions suggests disseminated disease.^4^ Unfortunately, the prognosis for metastatic melanoma is poor with 5 years survival rate estimated to be between 5–19% and median overall survival of 5.3 months; the prognosis is further influenced by location and the number of metastases.^26^ However, in case of the patient described in this report, muscle lesion was the only found metastasis with no other lesions seen on the staging CT, which is an unusual presentation of muscle metastasis. Furthermore, the lesion was assessed to be resectable and patient was referred to plastic surgery; additional treatment option for this patient will include adjuvant chemotherapy or radiotherapy in order to improve prognosis.

## Conclusion

Skeletal muscle metastases from melanoma primary are very rare and the differential diagnosis of sarcoma must be carefully considered when planning further investigations *e.g*. biopsy. Although melanoma metastases typically appear as hyperintense on *T*_1_-weighted MR imaging, the radiological appearances are highly variable and histopathological examination is necessary to confirm the diagnosis. Prognosis for metastatic melanoma is often quite poor, especially as muscle metastases are commonly a late event in disseminated disease.

## Learning points

- Breast Clinic offers an accessible service and as such plays an important in investigating non-breast pathologies including axillary and upper arm masses.
- Cooperation between different specialties, in particular input of radiologists at the MDT meetings, is vital in planning investigations of intramuscular lesions (*e.g.,* deciding on the type of biopsy performed) and reaching the diagnosis.
- Muscle metastases are associated with variable appearances on imaging, including MRI.
